# Expression profiles of the lncRNA antisense GAS5-AS1 in colon biopsies from pediatric inflammatory bowel disease patients and its role in regulating sense transcript GAS5

**DOI:** 10.1007/s00431-023-05403-4

**Published:** 2024-01-10

**Authors:** Debora Curci, Martina Franzin, Giulia Zudeh, Matteo Bramuzzo, Sara Lega, Giuliana Decorti, Gabriele Stocco, Marianna Lucafò

**Affiliations:** 1 Department of Advanced Translational Diagnostics, Institute for Maternal and Child Health IRCCS “Burlo Garofolo”, 34137 Trieste, Italy; 2 Department of Pediatric Gastroenterology, Institute for Maternal and Child Health IRCCS “Burlo Garofolo”, 34137 Trieste, Italy; 3 Department of Medicine Surgery and Health Sciences, University of Trieste, 34149 Trieste, Italy; 4 Department of Life Sciences, University of Trieste, 34127 Trieste, Italy

**Keywords:** Inflammatory bowel disease, Long non-coding RNA, Growth arrest-specific transcript 5, Antisense, Inflammation

## Abstract

The long non-coding RNA (lncRNA) growth arrest-specific transcript 5 (GAS5) level was demonstrated as involved in pediatric inflammatory bowel disease (IBD) pathogenesis. Since its antisense transcript GAS5-AS1 has never been investigated in IBD, this study aims to detect whether GAS5-AS1 and GAS5 levels are related to IBD clinical parameters and investigate their correlation in vitro. Twenty-six IBD pediatric patients were enrolled; paired inflamed and non-inflamed intestinal biopsies were collected. We evaluated GAS5 and GAS5-AS1 levels by real-time PCR. The role of GAS5 and GAS5-AS1 was assessed in vitro by transient silencing in THP1-derived macrophages. GAS5-AS1 and GAS5 levels were associated with patients’ clinical parameters; GAS5-AS1 expression was downregulated in inflamed tissues and inversely correlated with disease activity. A positive correlation between GAS5-AS1 and GAS5 levels was observed in non-inflamed biopsies. On THP1-derived macrophages, a reduced amount of both GAS5-AS1 and GAS5 was observed; accordingly, matrix metalloproteinase (MMP) 9 was increased. After GAS5-AS1 silencing, a downregulation of GAS5 was found, whereas no effect was detected on GAS5-AS1 after GAS5 silencing.

*    Conclusion*: This study provided for the first time new insights into the potential role of GAS5-AS1 in IBD. GAS5-AS1 modulates GAS5 levels in vitro and may serve as a potential IBD diagnostic biomarker.

**Table:** 

**What is Known:** *• GAS5 is involved in regulating intestinal MMP-2 and MMP-9 in pediatric patients with IBD*; *• GAS5-AS1 has never been investigated in the context of IBD*; *• GAS5-AS1 regulates the expression of GAS5, increasing its stability in tissues and in vitro cell models of cancer.*	
**What is New:** *• GAS5-AS1 correlated with GAS5 and IBD clinical parameters*; *• GAS5-AS1 can modulate GAS5 levels in macrophages*; *• GAS5-AS1 may serve as potential IBD diagnostic biomarker.*

**What is Known:**

*• GAS5 is involved in regulating intestinal MMP-2 and MMP-9 in pediatric patients with IBD*;

*• GAS5-AS1 has never been investigated in the context of IBD*;

*• GAS5-AS1 regulates the expression of GAS5, increasing its stability in tissues and in vitro cell models of cancer.*

**What is New:**

*• GAS5-AS1 correlated with GAS5 and IBD clinical parameters*;

*• GAS5-AS1 can modulate GAS5 levels in macrophages*;

*• GAS5-AS1 may serve as potential IBD diagnostic biomarker.*

## Introduction

Inflammatory bowel diseases (IBD) are a group of chronic disorders affecting the gastrointestinal tract and comprise Crohn’s disease (CD) and ulcerative colitis (UC), which can be distinguished by symptomatology, disease location, and type of inflammation [1]. To date, the aetiology of IBD remains unknown. However, several features such as genetic susceptibility, aberrant immune response of the host and exposure to environmental factors are considered to contribute to the onset of the disease [2]. IBD is most often diagnosed in adolescence, with increasing incidence in the pediatric population.

The incidence of IBD varies considerably both within and between geographic regions. In particular, Europe and North America have reported the highest incidence rates. Among factors involved in IBD pathogenesis, genetic factors seem to have an important role. To date, more than 200 IBD-associated risk alleles have been identified using GWAS studies, and 71 IBD-associated genetic risk alleles have been shown to differ according to geographic regions [3]. However, no associations were found between GAS5 or GAS5-AS1 genetic variants and IBD between different populations.

Children with IBD usually present more extensive anatomic involvement, more severe disease course, and often require stronger treatment compared to patients with adult disease onset [4, 5]. The implications of IBD are of particular importance in children because of the potential negative effects on growth and development [6]. Nowadays, a curative therapy does not exist, and medical treatment aims at inducing clinical remission by controlling inflammation and symptoms and maintaining remission to prevent relapses.

In this context, exploring molecular mechanisms associated with IBD would be necessary to better understand this disease’s pathogenesis and thus identify novel therapeutic targets.

Growing evidence validates the fundamental role of epigenetic factors, such as long non-coding RNAs (lncRNAs), in regulating several pathological conditions and inflammatory diseases, including IBD [7–10]. In particular, lncRNAs are mRNA transcripts, have a size of more than 200 nucleotides, are not translated into proteins, and handle several pathophysiological processes through the interaction with chromatin-modifying proteins, acting as transcriptional enhancers or interfering at post-translational level [7, 8, 11].

Noteworthy, lncRNAs are differentially expressed in IBD, suggesting their influence on the course of inflammation [12, 13]. For instance, the lncRNA growth arrest-specific transcript 5 (GAS5), one of the most studied, is downregulated in inflamed colonic tissues of pediatric patients with IBD and modulates the expression of the matrix metalloproteinases (MMPs) 2 and 9, involved in the pathogenesis of IBD [14].

GAS5-AS1 is a lncRNA located on chromosome 1q25.1, and it is the GAS5 antisense. Interestingly, these lncRNAs present a complementary sequence of 40 nucleotides, determining their possible physical interaction [15]. GAS5-AS1 has been associated with developing and progressing different types of cancers, such as non-small cell lung cancer, hepatocellular carcinoma, and gliomas [16–18]. However, it has never been investigated in the context of IBD. Interestingly, GAS5-AS1 regulates the expression of GAS5, increasing its stability in tissues and in vitro cell models of cervical cancer [19].

In this investigation, we evaluate the contribution of GAS5-AS1 in colon biopsies of IBD pediatric patients and explore the correlation between GAS5-AS1 and GAS5 levels on THP-1 derived macrophages, an immune system in vitro model.

## Results

### Patients

Twenty-six children with IBD (CD 12; UC 14) were enrolled since October 2013 to November 2016. In particular, 20 patients were enrolled at IBD diagnosis and were not yet in treatment, for 6 patients biopsies were collected not at diagnosis (median time since diagnosis 2.25 years, IQR 2–4.97), during aminosalicylates therapy. The demographic and clinical characteristics of the population are shown in Table 1.

**Table 1:** Demographic and clinical characteristics of the patients

**Patients (*n*)**	26
**Biopsies collected at IBD diagnosis (%)**	20 (76.9%)
**Biopsies collected after diagnosis (%) **	6 (23.1 %)
**Age (years, median, IQR) at time of sample collection**	12, 6.3–18
**Male**	11 (42.3%)
**CD**	12 (46.2%)
**UC**	14 (53.8%)
**C reactive protein (mg/L, median, IQR)**	3.5 (1–27.3)
**Faecal calprotectin (µg/g, median, IQR)**	1809.0 (1398.2–2119.7)
**BMI (kg/m**^**2**^**, median, IQR)**	19 (16–22.65)
***Clinical Score:***	
** PCDAI (median, IQR)**	31.3, 7.5–55
** PUCAI (median,IQR)**	30.0, 10–75

### GAS5-AS1 expression in mucosal biopsies of pediatric IBD patients

**Fig. 1:**
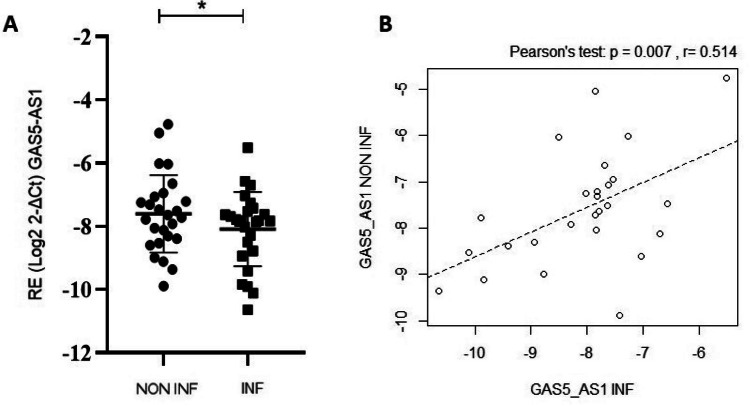
Growth arrest-specific transcript 5 antisense 1 (GAS5-AS1) levels in colon biopsies of 26 inflammatory bowel disease (IBD) pediatric patients. **A** GAS5 and GAS5-AS1 gene expression levels were evaluated in each patient's inflamed (INF) and non-inflamed (NON-INF) tissues and normalized using the RPLP0 gene. Relative expression (RE) values were expressed as Log_2_2^-ΔCt^ (paired *t*-test * *p* < 0.05). **B** Pearson correlation between GAS5-AS1 levels in inflamed (INF) and non-inflamed (NON-INF) tissues (Pearson’s test *p* = 0.007)

GAS5-AS1 gene relative expression was evaluated in inflamed and non-inflamed biopsies of IBD patients, revealing a statistically significant downregulation in inflamed compared to non-inflamed mucosa (*t*-test *p* = 0.046, Fig. 1A). Considering the impact of IBD type on GAS5-AS1 levels, a statistically significant downregulation in inflamed mucosa compared to non-inflamed one was observed only for UC patients (*t*-test *p* = 0.018) but not for CD (*t*-test *p* = 0.629), where however a trend was still present. Also, a significant positive correlation between GAS5-AS1 levels in inflamed and non-inflamed biopsies was detected (Pearson’s test *p* = 0.007, Fig. 1B).

### Correlation of GAS5-AS1 expression in mucosal biopsies with clinical parameters

**Fig. 2:**
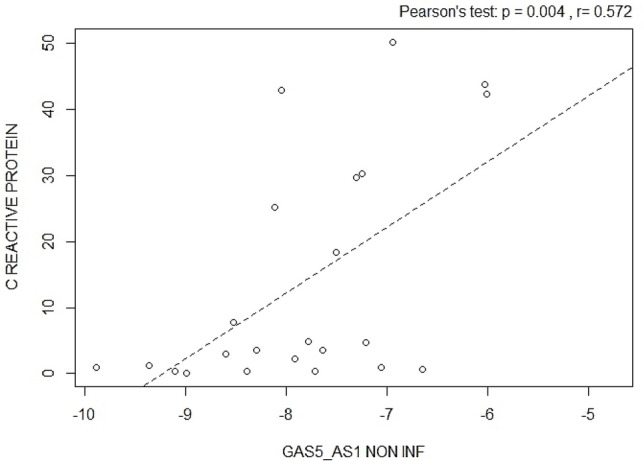
Pearson correlation between GAS5-AS1 and C reactive protein in non-inflamed colon biopsies (Pearson’s test *p* = 0.004)

GAS5-AS1 and GAS5 levels were measured in inflamed and non-inflamed colon tissues and evaluated with the clinical parameters, as reported in Table 1. Significant positive correlations were found between GAS5-AS1, measured in non-inflamed biopsies, and serum C reactive protein (Pearson’s test *p* = 0.004, Fig. 2); conversely, GAS5 resulted not significantly correlated with serum C reactive protein both in non-inflamed and inflamed biopsies (Pearson’s test *p* = 0.121 and *p* = 0.268, respectively).

**Fig. 3:**
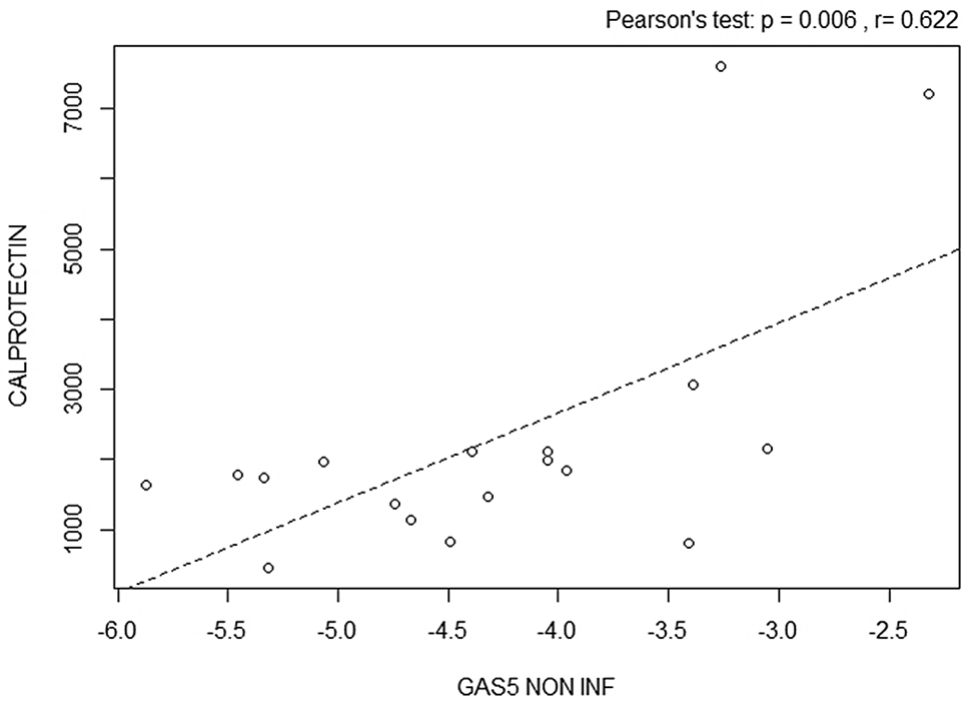
Pearson correlation between GAS5 in non-inflamed (NON-INF) tissues and fecal calprotectin (Pearson’s test *p* = 0.006)

No correlations were found between GAS5-AS1 and fecal calprotectin measured in both inflamed and non-inflamed biopsies (Pearson’s test *p* = 0.918 and *p* = 0.265), but a positive correlation was found between GAS5, measured in non-inflamed biopsies, and fecal calprotectin (Pearson’s test *p* = 0.006, Fig. 3). Moreover, in inflamed biopsies, a positive trend was detected (Pearson’s test *p* = 0.058).

**Fig. 4:**
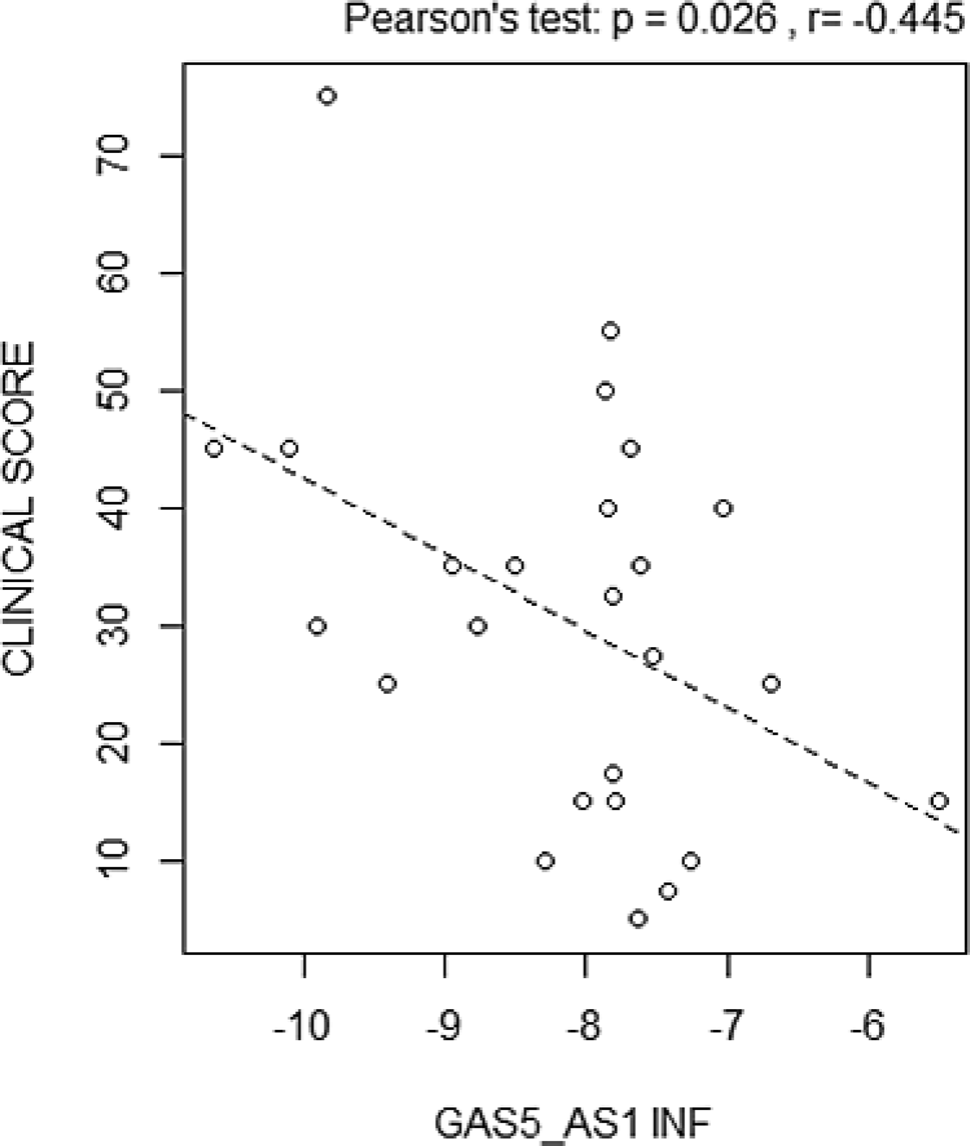
Correlation between GAS5-AS1 evaluated in inflamed (INF) tissues and clinical score (Pearson’s test *p* = 0.026)

GAS5-AS1 also correlated negatively with the disease activity scores in inflamed colon samples (Pearson’s test *p* = 0.026, Fig. 4).

No correlations were found between BMI and lncRNA levels measured both in non-inflamed and inflamed biopsies (Pearsons’s test *p* > 0.05).

### Correlation of GAS5-AS1 and GAS5 expression in mucosal biopsies of pediatric IBD patients

**Fig. 5:**
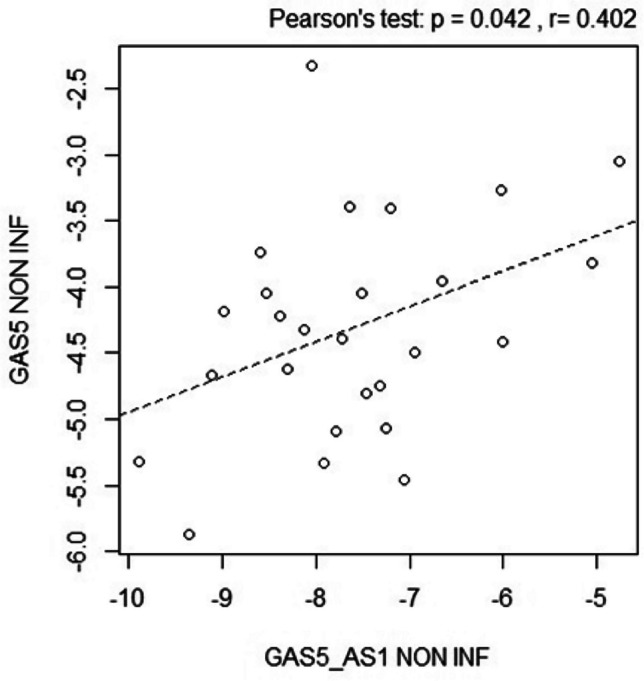
Correlation between GAS5-AS1 and GAS5 levels evaluated in non-inflamed (NON-INF, Pearsons’s test *p* = 0.04) tissues

Since a direct interaction between GAS5-AS1 and GAS5 has been described [19, 21], the correlation of their expression was determined in biopsies of IBD patients. As reported in Fig. 5, GAS5-AS1 was positively correlated with GAS5 expression in non-inflamed tissues (Pearsons’s test *p* = 0.042), but no significant association was found in the inflamed area (Pearsons’s test *p* = 0.826).

### Correlation of GAS5-AS1 and GAS5 expression in THP-1-derived macrophages

**Fig. 6:**
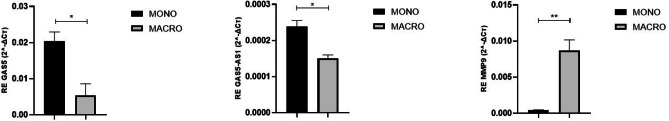
GAS5, GAS5-AS1, and MMP9 expression in THP-1 monocyte and in THP-1 derived macrophages, an inflamed cellular model. *T*-test: *p* < 0.05 was considered statistically significant

In order to deeper investigate the possible role of inflammation in the expression of GAS5-AS1 and GAS5, a comparison in the expression level of GAS5-AS1, GAS5, and the inflammatory modulator MMP9 in THP1 monocytes and in the inflammatory model THP1 macrophages cells was performed, showing a reduced amount of both GAS5-AS1 (*t*-test *p* = 0.035) and GAS5 (*t*-test *p* = 0.030) in macrophages; accordingly, MMP9 was increased (*t*-test *p* = 0.004, Fig. 6).

**Fig. 7:**
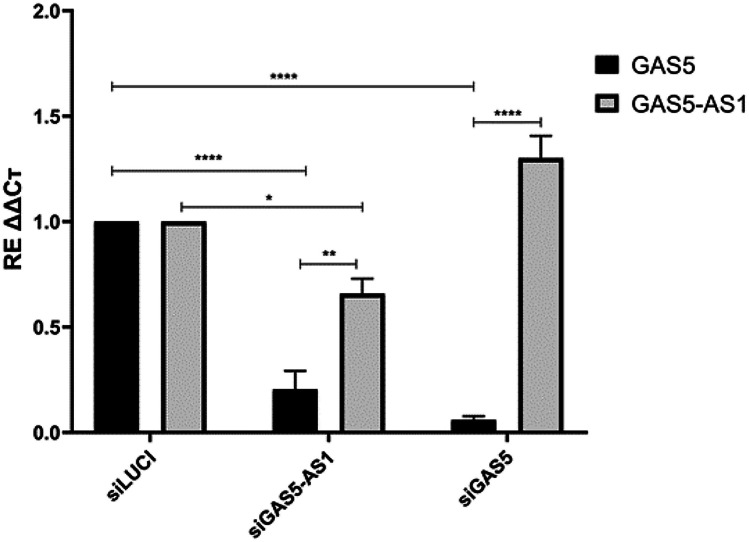
GAS5-AS1 and GAS5 expression correlate with siLUCI control in THP-1-derived macrophages after transient GAS5-AS1 and GAS5 silencing. Two-way ANOVA: *p* < 0.0001; Tukey’s multiple comparisons test:****p < 0.0001, **p = 0.003, *p = 0.023

To further investigate the correlation between GAS5-AS1 and GAS5, mRNA expression of these lncRNAs, compared to the siLUCI control, was evaluated on THP-1-derived macrophages after the transient silencing of GAS5-AS1 (*t*-test *p* = 0.023) and GAS5 (*t*-test *p* =  < 0.0001), respectively. As reported in Fig. 7, GAS5-AS1 transient silencing led to a reduction in GAS5 expression in comparison with siLUCI (*t*-test *p* = 0.0001). In contrast, the GAS5 silencing did not affect the GAS5-AS1 expression compared to control.

## Discussion

Previous studies demonstrated lncRNA GAS5-AS1 as a tumor suppressor able to affect cancer pathogenesis, metastasis, prognosis, and survival [18, 19, 22]; however, no studies have evaluated its possible involvement in non-tumoral conditions, such as IBD. Our results demonstrated that the expression of GAS5-AS1 in pediatric patients with IBD was lower in inflamed than non-inflamed tissue. Several studies demonstrated that protein family of histone deacetylases (HDACs) influence inflammation and intestinal epithelium barrier function, indicating their possible role in IBD and represent promising therapeutic targets that could help to control inflammation and protect the intestinal barrier integrity. In particular, HDACs are involved in different inflammatory pathways through the interaction with key inflammatory regulators [23]. Epigenetic modifications, such as histone modification, may be involved in GAS5 and GAS5-AS1 transcriptional deregulation, although the underlying mechanism should be further studied. Jiajie Tu and colleagues reported that the promoter sequence of GAS5 is more conserved than that of the gene body across different species, and its genomic locus is enriched with epigenetic marks related to transcriptional activation indicating that GAS5 transcription appears to be tightly regulated by epigenetic mechanisms [24]. It is known that the knockdown of HDAC1 or HDAC3 enhances in vitro expression of GAS5-AS1 and HDAC inhibitors significantly induced GAS5-AS1 in a dose-dependent manner [18]. Given that a reduction in the percentage of histone-3 lysine-27 acetylation (H3K27ac) positive cells was described in actively inflamed IBD biopsies [25], the downregulation of GAS5-AS1 observed in our cohort could depend on these epigenetic mechanisms. In this context, HDAC inhibitors already tested for their anti-inflammatory properties in mouse dextran sulfate sodium (DSS)-induced colitis models may also be effective due to their ability to increase GAS5-AS1 levels. Recent works have demonstrated that HDAC inhibitors can attenuate the inflammatory profile in DSS-induced colitis models by suppressing local secretion of pro-inflammatory cytokines and chemokines and also by suppressing mobilization and accumulation of inflammatory cells [26]. Further studies should be performed to confirm the role of histone modification in regulating GAS5-AS1 expression in IBD patients.

Interestingly, a significant negative correlation between GAS5-AS1 levels and patient’s clinical scores was detected in the inflamed intestinal samples, indicating that high GAS5-AS1 levels could be associated with a clinical outcome improvement. In addition, fecal calprotectin and C reactive protein correlated with GAS5 and its antisense levels, suggesting their possible involvement in the intestinal inflammation and disease progression. In recent years, similar results were found for GAS5, which was reduced in inflamed colon biopsies deriving from IBD pediatric patients compared to the non-inflamed ones; GAS5 downregulation was also associated with the up-regulation of matrix metalloproteinases (MMPs) [14], which are responsible for the extracellular matrix (ECM) turnover and also for the regulation of ECM composition in terms of inflammatory chemokines, cytokines and growth factors[27].

GAS5 and GAS5-AS1 present around 40 complementary nucleotides, responsible for their physical interaction at the 3’ terminal [21], and a previous study showed that GAS5-AS1 overexpression prolonged the half-life of GAS5, whereas GAS5-AS1 silencing reduced the half-life of GAS5 in cervical cancer cells [21]. As GAS5 modulates the expression of MMPs in inflamed tissues [14], both GAS5-AS1 and GAS5 levels could represent possible biomarkers of the inflammatory status, related to an active disease score. Moreover, GAS5-AS1 may be useful in the clinic for steroid therapy, thanks to its ability to modulate GAS5, which is implicated in steroid response through the binding to the glucocorticoid receptor [28, 29].

Consistently, our data indicated a direct correlation between GAS5 and GAS5-AS1 levels in non-inflamed IBD colonic biopsies; however, this trend was missed in the inflamed samples, indicating that inflammation may affect their expression or stability.

The correlation between these lncRNAs was investigated on THP-1-derived macrophages, representing an in vitro model of the immune cells. Our results proved that the silencing of GAS5-AS1 led to a downregulation of GAS5; on the contrary, no effect on GAS5-AS1 amount was detected after GAS5 transient silencing, confirming that GAS5-AS1 can modulate GAS5 levels in macrophages similarly to what was already demonstrated in cancer cells [19]. To further evaluate the possible inflammation role in the amount of these lncRNAs, we tested GAS5-AS1, GAS5, and the inflammatory modulator MMP-9 in THP1 monocytes and the inflammatory model THP1 macrophages. Monocytes and macrophages play pivotal roles in the innate immune response, and their inappropriate activation can induce sustained inflammation resulting in autoimmune and inflammatory diseases, such as IBD [30, 31]. Interestingly, we found higher MMP9 levels and a reduction of both GAS5-AS1 and GAS5 in macrophages compared to monocytes, demonstrating similar trends between these lnRNAs.

Ghorbaninejad M. and colleagues used an established co-culture system including intestinal epithelial Caco-2 cells and RAW264.7 macrophage cells to demonstrate the downregulation of GAS5 in inflamed enterocyte-like cells [32], confirming the trend observed in our patients.

The methylation at the sixth position of an adenine base in an RNA molecule, called N6-methyladenosine (M6A), is the most prevalent modification in several classes of RNAs, including mRNA and lncRNAs, and it is considered a post-transcriptional regulator of physiological and pathological processes [21]. M6A modification in RNA can be modulated by different RNA demethylases, such as the alpha-ketoglutarate-dependent dioxygenase alkB homolog 5 (ALKBH5). Mechanistically, GAS5-AS1 binds GAS5 and increases its stability through the interaction with ALKBH5, altering the GAS5 M6A modification level [13]. Additionally, the interaction between GAS5-AS1 and GAS5 is also mediated by YTH N6-Methyladenosine RNA binding protein F2 (YTHDF2), an enzyme able to recognize the M6A modification in different classes of RNA, regulating their half-life [33, 34]. It was demonstrated that the M6A RNA modification machinery plays a role in maintaining intestinal homeostasis, and its disruption may contribute to developing intestinal inflammatory conditions and colonic cancers [35–37]. It was previously demonstrated that UC patients with higher m6A methylation presented an increased expression of both ALKBH5 and YTHDF2, which could be also considered IBD biomarkers important to determine patients’ outcome [38, 39]. Therefore, it would be interesting to evaluate GAS5-AS1 and GAS5 M6A modification levels and the amount of both ALKBH5 and YTHDF2 in colonic biopsies of IBD patients to further investigate the molecular mechanisms at the basis of the different correlation between GAS5 and its GAS5-AS1 detected in the inflamed and non-inflamed colon samples. This consideration may explain the observed enhanced or diminished transcripts abundance of these lncRNAs in our IBD pediatric patient’s cohort.

This study presented some limitations regarding the relatively small size of the cohort. However, this is the first study to consider the role of GAS5-AS1 expression in colon biopsies from pediatric inflammatory bowel disease patients, a very relevant and important tissue to investigate new players in IBD pathophysiology. Future investigations should be assessed in a larger cohort to profoundly investigate the molecular mechanisms undergoing the role of GAS5-AS1 in the IBD pathogenesis, highlighting the possible role of GAS5-AS1 as a potential diagnostic biomarker and its possible implications in the development of innovative IBD treatment strategies, particularly to guide glucocorticoids (GC) therapy and to determine steroids effectiveness, thanks to the ability of GAS5-AS1 to modulate GAS5, which is implicated in GC response. In addition, it will be essential to evaluate the role of these lncRNAs in different cell types present in the colonic biopsies to further understand the possible contribution of GAS5-AS1 and GAS5 in the inflammatory modulation.

## Materials and methods

### Clinical samples

Twenty-six IBD pediatric patients were enrolled at the Gastroenterology Department of the Pediatric Clinic of IRCCS Burlo Garofolo in Trieste. For each patient, two colonic biopsies of inflamed and non-inflamed areas were collected and immediately stored in TRIzol® reagent (Thermo Scientific, Carlsbad, CA, USA). Inflamed and non-inflamed biopsies were distinguished by clinician based on macroscopically observation of the colon during colonoscopy. Biopsies of non-inflamed tissues were collected from areas proximal to the inflamed ones.

The study was conducted following the Declaration of Helsinki, and the Institutional Ethics Committee approved the protocol (Protocol number 2198; September 17th 2013). All subjects and parents signed informed consent before participating in the study. Clinical disease activity was assessed at the time of biopsy collection, using pediatric Crohn’s disease activity index (PCDAI) and pediatric ulcerative colitis activity index (PUCAI) for CD and UC patients, respectively [14]. In addition, levels of inflammatory biomarkers serum C reactive protein (mg/L) and fecal calprotectin (µg/g) were evaluated at the same time as the colonoscopy. Remission was defined as a total PCDAI or PUCAI score ≤ 10.

### Total RNA isolation

Total RNA was extracted from biopsies and cells using TRIzol® reagent (Thermo Scientific, Carlsbad, CA, USA) according to the manufacturer’s instructions. The RNA concentration and purity were calculated by NanoDrop instrument (NanoDrop 2000, EuroClone, Milan, Italy).

### Quantitative real-time PCR

*GAS5-AS1* and *GAS5* genes mRNA expression was evaluated by real-time RT-PCR TaqMan® analysis using the CFX96 real-time system-C1000 Thermal Cycler (Bio-Rad Laboratories, Hercules, CA, USA). The reverse transcription reaction was carried out with the High Capacity RNA-to-cDNA Kit (Applied Biosystem, Foster City, CA, USA), and the real-time PCR was performed in triplicate using the TaqMan® Gene Expression Assay, according to the manufacturer’s instructions. The thermal cycling conditions for TaqMan® assays were as follows: 2 min at 50 °C and 10 min at 95 °C followed by 40 cycles at 95 °C for 15 s and 60 °C for 60 s. The expression levels were evaluated using the comparative Ct method (2^-ΔCt^ or 2^-ΔΔCt^). *GAS5-AS1* and *GAS5* mRNA expression values were normalized utilizing the housekeeping *RPLP0* gene.

### THP-1 cell culture and silencing of GAS5 and GAS5-AS1

The THP-1 monocytic cells (ATCC, TIB-202) were seeded in 24-well plates at 0.5 × 10^5^ cells/well and were grown in RPMI 1640 medium (EuroClone®, Milan, Italy) for 24 h. THP-1 cells were differentiated into macrophages using 5 ng/mL of phorbol-12-myristate 13-acetate (PMA, Sigma-Aldrich, Saint Louis, MO, USA) for 48 h. Thirty nM of silencer Select GAS5, GAS5-AS1, and the Negative Control siRNAs (Thermo Scientific, Carlsbad, CA, USA) were transfected by employing Lipofectamine RNAiMAX Transfection Reagent (Thermo Scientific, Carlsbad, CA, USA) following the manufacturer’s guidelines and instruction. The transfection was repeated every 24 h for a total of 72 h. TRIzol® reagent (Thermo Scientific, Carlsbad, CA, USA) was used for RNA isolation.

### Biostatistics

Statistical analyses were performed with GraphPad Prism and R software. One- and two-way ANOVA with Tukey’s multiple comparison tests were applied for the analysis of gene expression. Shapiro test assessed the normalization of the distribution of the data. Data were normalized by logarithmic transformation. Paired *t*-test or Pearson correlation was involved in analyzing categorical and continuous independent variables, respectively. *P*-values < 0.05 were considered statistically significant. In vitro analyses were performed at least three times.

## Conclusions

These data revealed that intestinal inflammation promoted a downregulation of GAS5-AS1 levels, which correlated negatively with the disease score, irrespective of patient sex and age. A positive correlation between GAS5 and GAS5-AS1 expression levels was detected only in non-inflamed samples, suggesting that during inflammation, epigenetic factors such as histone acetylation and M6A RNA modification, could reduce GAS5-AS1 level, which is responsible for GAS5 stability maintenance. The *in vitro* results confirmed that GAS5-AS1 was a regulator of GAS5 expression in macrophages, underlying the importance of GAS5-AS1 amount in the immune system.

This is a pilot study, and for this reason, further analyses will be necessary to better clarify the role of both lncRNAs in IBD pathogenesis and understand their clinical value in terms of potential diagnostic and prognostic markers and as therapeutic targets.

## Data Availability

The raw data supporting the conclusions of this article will be made available by the authors without undue reservation.

## Abbreviations

IQR: Range interquartile
CD: Crohn’s disease
UC: Ulcerative colitis
BMI: Body mass index
PCDAI: Pediatric Crohn’s disease activity index
PUCAI: Pediatric ulcerative colitis activity index [20]
